# Clinical outcomes of a new local CD19 CAR-T cell therapy for patients with relapsed or refractory acute lymphoblastic leukemia and non-Hodgkin lymphoma in Malaysia

**DOI:** 10.1038/s41409-025-02629-8

**Published:** 2025-05-27

**Authors:** S. Fadilah Abdul Wahid, Nor Azimah Ismail, Muhammad Firdaus Md Fauzi, Mohd Razif Mohd Idris, Mohd Syahrir Yusop, Timothy Lim, Nor Asiah Muhamad

**Affiliations:** 1 Pusat Terapi Sel (PTS), Hospital Canselor Tuanku Muhriz Universiti Kebangsaan Malaysia (HCTM-UKM), Kuala Lumpur, Malaysia; 2 Department of Molecular Imaging & Nuclear Medicine, Hospital Canselor Tuanku Muhriz, Universiti Kebangsaan Malaysia (HCTM-UKM), Kuala Lumpur, Malaysia; 3 Plutonet Sdn Bhd, Cyberjaya, Selangor Malaysia; 4 Sector for Evidence-based Healthcare, National Institutes of Health, Ministry of Health Malaysia, Putrajaya, Malaysia

**Keywords:** Acute lymphocytic leukaemia, B-cell lymphoma

## To the editor:

CD19-targeting chimeric antigen receptor T-cell (CAR-T) therapy has demonstrated remarkable efficacy in treating relapsed/refractory (R/R) B-cell acute lymphoblastic leukemia (B-ALL) and non-Hodgkin lymphoma (B-NHL) [1–5]. Two Food and Drug Administration approved CAR-T products are commercially available for B-ALL, and four for B-NHL. However, its availability for Malaysian patients remains limited due to high costs and the absence of approved products. Plutonet Sdn Bhd has introduced locally manufactured CD19-CAR-T cells to overcome these challenges, providing an affordable and accessible solution for R/R B-ALL and B-NHL patients in Malaysia who have failed standard therapies.

We report the clinical outcomes of 30 patients treated with a new local CAR-T cell product developed locally. Two open-label, single-arm prospective phase II trials were conducted at Pusat Terapi Sel, Hospital Canselor Tuanku Muhriz, Universiti Kebangsaan Malaysia (UKM) from 2019 to 2025 in R/R B-ALL and B-NHL treated with autologous CD19-41BB CAR-T cells (PP CAR-T). PP CAR-T characteristics, study methods, and enrollment details are described in Supplementary Materials.

**Fig. 1:**
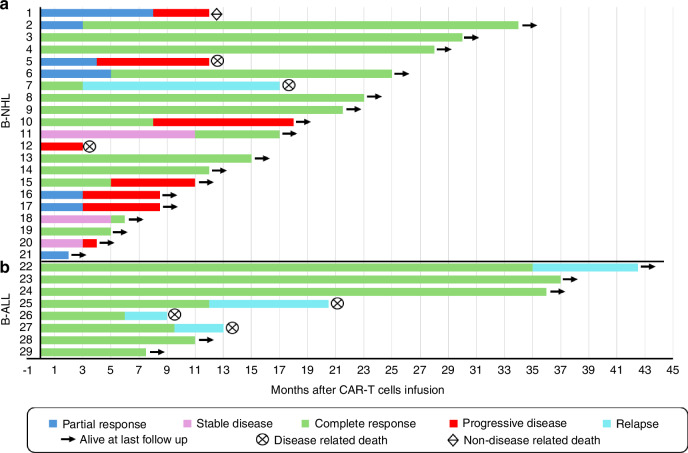
Overall treatment response in 29 patients (B-ALL; 8; B-NHL: 21) treated with PP CD19 CAR-T cell therapy. **a** B-NHL, **b** B-ALL.

A total of 73 patients were enrolled, of whom 56 proceeded to apheresis (Fig. 1). PP CAR-T cells were manufactured for 31 patients and ultimately administered to 30 patients (41% of the total cohort). Approximately one-quarter of enrolled patients (*n* = 17) did not undergo apheresis due to rapid disease progression leading to death (*n* = 8) or inadequate T-cell fitness as determined by a failed T-cell assimilation test (*n* = 7). Among those who underwent apheresis, the main reason for not receiving CAR-T therapy was cancer-related mortality (*n* = 19; 6 B-ALL, 13 B-NHL), with only one case of manufacturing failure (3.2%). These high attrition rates (58.9%) underscore the critical importance of early referral—before disease progression precludes eligibility for clinical trial participation—and timely T-cell collection to preserve T-cell fitness and improve the likelihood of CAR-T treatment delivery.

The median time from apheresis to start of culture was 61.5 days (11–241), while the median time for cell culture was 14 days. The median dose of CAR-T cells infused were 2 × 10^6^/kg (1.0–2.85), in which most (24 of 30) patients received fresh CAR-T cells.

Safety and efficacy were assessed at the data cutoff of March 31, 2025. Results were compared with those of commercial 4-1BB co-stimulated CAR T-cell products, specifically Tisagenlecleucel (Tisa-cel). The median follow-up period for the overall cohort was 12.5 months (0.5–42.5 months).

Table 1 shows patient and disease baseline characteristics and outcomes of PP CAR-T therapy.

**Table 1:** Patient baseline characteristics and PP CAR-T cell treatment outcomes.

Parameters	B-ALL	B-NHL	Total patients
	*n* (%)	*n* (%)	*n* (%)
Subjects evaluable for safety, efficacy	8.8	22.21	30.29
Median age (range)	29 years (15–50)	56 years (26–72)	47.5 years (15–72)
Male	3 (37.5)	14 (63.6)	17 (56.7)
ECOG at study entry	0-1: 8 (100)	0–1: 19 (86.4) 2–3: 3 (13.6)	0–1: 27 (90.0) 2–3: 3 (10.0)
Tumor subtype	Pre B -ALL Ph-positive: 2 (25.0)	DLBCL:14 (63.6) PBMCL: 5 (22.7) FL: 1 (4.55) MCL: 1 (4.55) High grade: 1 (4.55) Double expressor: 1 (4.55) Triple expressor: 4 (18.2)	
Tumor cell of Origin	NR	GCB: 3 (13.6) ABC: 4 (18.2)	
Tumor stage	NR	Stage 2: 6 (27.3) Stage 3: 6 (27.3) Stage 4: 10 (45.5)	
Serum LDH (>200 U/L)	NR	11 (50.0)	
EM, EN and BM involvement	EM: 0	EN: 11 (50.0) BM: 3 (13.6)	
Bulky disease (more than 10 cm)	NR	2 (9.1%)	
Prior Lines of treatment (median, range) (number of patients, %)	3 (2–3) 1–2:2 (25.0%) 3–4: 6 (75.0%)	2.5 (1–4) 1-2: 11 (50.0%) 3–4: 11(50.0%)	3 (1–4) 1–2: 13 (43.3%) 3–4: 17 (56.7%)
History of primary refractory disease	3 (37.5%)	Primary refractory 11 (50.0) Relapse 11 (50.0)	
Disease status at enrollment	>5% blast in BM 7 (87.5)	PR 1 (7.1) PD 12 (85.7) SD 1 (7.1)	
Prior HSCT			
Allogeneic	4 (50.0)	0	4 (13.3)
Autologous	1 (12.5)	6 (27.3)	7 (23.3)
Bridging therapy	5 (62.5)	16 (72.7)	21 (70)
CAR-T cell dose × 10^6^/kg, % viability (median, range)	2 (1.2–2.50), 84.8 (70.0–93.6)	2 (1.0–2.85), 85.5 (73.5–94.8)	2 (1.0–2.85), 85.5 (70.0–94.8)
Median follow-up (median, range)	16.8 (7.5–42.5 months)	12 (0.5–34 months)	12.5 (0.5–42.5 months)
CRS:			
All grades,	6 (75.0)	8 (36.4)	14 (46.7)
Grade ≥ 3	0	1 (4.5)	1 (3.3)
ICANS:			
All grades,	None	None	1 (3.3)
Grade ≥ 3		1 (4.5)	1 (3.3)
Cytopenia Grade ≥3	8 (100)	19 (86.4)	27 (90.0)
Febrile neutropenia	3 (37.5)	4 (18.2)	7 (23.3)
COVID infection	2 (25.0)	1 (7.1)	3 (10.0)
Infection (non COVID)			
Grade 3	1 (12.5)	4 (18.2)	
Grade 4	1 (12.5)	1 (4.5)	
B cell aplasia and serum IgG	8 (100)	18 (81.8)	26 (86.7)
level below 400 mg/dL	4 (40)	6 (27.3)	10 (33.3)
Immunoglobulin replacement therapy	None	7 (33.3)	
Best overall outcome			
CR	8 (100) MRD negative CR = 7 (87.5)	14 of 21 (66.7)	
PR	NA	5 of 21 (23.8)	
SD	NA	1 of 21 (4.8)	
PD	NA	1 of 21 (4.8)	
Ongoing response	4 (50.0) at med follow-up 16.8 months	12 of 21 (57.1) at med follow-up 12 months	
CR	4 (50.0)	11 of 21(52.4) 11 of 19	
PR	NA	1 of 21 (4.8)	
DoR, (median, IQR)	35 (13.6,56.3)	Not reached	
Median OS	Not reached	Not reached	

Data are expressed as *n* (%) unless otherwise indicated.

Minimal residual disease (MRD) negativity is the absence of detectable leukemic cells with immunophenotypic aberrancies at a sensitivity of 10−4 or an undetectable bcr-abl transcript [6].

*ABC* activated B cell-like type, *B-ALL* B acute lymphoblastic leukemia, *B-NHL* B-cell non-Hodgkin lymphoma, *ECOG* Eastern Cooperative Oncology Group performance status, *DLBCL* diffuse large B-cell lymphoma, *PBMCL* primary mediastinal large B-cell lymphoma, *FL* follicular Lymphoma, *NR* not relevant, *LDH* lactate dehydrogenase, *EM* extramedullary, *EN* extranodal, *BM* bone marrow, *HSCT* hematopoietic stem cell transplantation, *CRS* cytokine release syndrome, *ICANS* immune effector cell-associated neurotoxicity syndrome, *OR* overall response rate, *CR* complete response rate, *GCB* germinal center B cell-like type, *MRD* minimal residual disease, *DoR* duration of response, *OS* overall survival, *PD* progressive disease, *NA* not applicable.

Among the eight patients with B-ALL, two were Philadelphia chromosome-positive (Ph+). Most of the B-ALL patients (75%) had primary chemorefractory disease. Three patients had received 3 to 4 prior lines of therapy, while four had undergone allogeneic hematopoietic stem cell transplantation (HSCT), and one had received an autologous HSCT.

Among the 22 patients with B-NHL, the majority were diagnosed with diffuse large B-cell lymphoma (DLBCL), including five cases classified as double- or triple-expressor lymphomas. Half of the patients had primary chemorefractory disease, and a similar proportion had received three to four prior lines of therapy. Six had previously undergone autologous HSCT. At baseline, elevated serum LDH levels were observed in 50% of patients, while 11 presented with extranodal involvement, and 3 had bone marrow infiltration. Further details on disease subtypes, median number of prior treatments, and other relevant disease and patient characteristics are summarized in Table 1.

## Safety profile of PP CAR-T therapy

The overall safety profile of PP CAR-T therapy was favorable, with the majority of adverse events (AEs) being grade I–II in severity. In the B-ALL cohort, cytokine release syndrome (CRS) occurred in 75.0% of patients, all of whom experienced only grade I–II events. These were effectively managed with tocilizumab and corticosteroids. This contrasts with prior studies of Tisagenlecleucel (Tisa-cel), where grade ≥3 CRS was reported in 46% of patients [1].

In the B-NHL group, CRS was observed in 46.7% of patients (14 of 22), including one grade 3 case and one fatal grade V CRS case attributed to delayed hospital admission and non-adherence to the study protocol. Despite these isolated severe cases, the overall incidence of grade ≥3 CRS in the B-NHL cohort remained low and was consistent with prior Tisa-cel data (4.5–23%) [3, 4].

Notably, no cases of immune effector cell-associated neurotoxicity syndrome (ICANS) were observed across our patient population. This represents a significant advantage over other commercial CAR-T therapies. In contrast, grade ≥3 ICANS has been reported in up to 13% of patients treated with Tisa-cel in B-ALL [1] and in 11% of B-NHL [3], highlighting a potentially lower neurotoxicity risk associated with PP CAR-T therapy.

Cytopenias are a well-documented and common complication following CAR-T therapy, often biphasic and multifactorial in nature [6]. Grade 3–4 cytopenias persisting beyond 30 and 90 days have been reported in 30–40% and 3–22% of patients, respectively, in those treated with commercial CAR-T products [3, 4, 7]. In our study, cytopenias were the most frequently reported AE in both cohorts, but were generally reversible and manageable with growth factors and transfusional support.

Grade ≥3 cytopenias occurred in all B-ALL and 86.4% of B-NHL patients. Febrile neutropenia occurred in 37.5% of B-ALL and 18.2% of B-NHL patients, and all cases resolved with antibiotic therapy. These rates are comparable to those reported for Tisa-cel, with febrile neutropenia occurring in 36% of B-ALL and 17% of B-NHL patients [1, 3].

Hypogammaglobulinemia was frequently observed; however, fewer than one-third of patients required intravenous immunoglobulin (IVIG) replacement, aligning with prior studies [7, 8]. IVIG administration was reserved for patients with a history of severe infections and serum IgG levels below 400 mg/dL. Importantly, despite the high incidence of cytopenias, the rate of severe infections remained low and responded well to supportive care.

## Efficacy of PP CAR-T therapy

The efficacy of PP CAR-T therapy compares favorably with that of approved CAR-T products reported in pivotal registration trials. In our B-ALL cohort (*n* = 8), all patients achieved a complete response (CR), with 87.5% (*n* = 7) attaining minimal residual disease (MRD)-negative status within 1–3 months following CAR-T infusion. These outcomes are comparable to those reported in a Chinese study utilizing a 4-1BB-based CAR-T construct (CR: 90%, MRD-negative: 85%) [2], as well as with Tisa-cel, which demonstrated a CR rate of 81% with MRD negativity [1].

At median follow-up durations of 16.8 and 12 months, respectively, sustained remission was maintained in 50% of PP CAR-T-treated B-ALL patients, comparable to 59% reported with Tisa-cel [1]. All responding patients in our study developed B-cell aplasia (Table 1), consistent with findings from previous studies using Tisa-cel [1].

Three MRD-negative patients experienced disease relapse at 6, 12, and 36 months post-infusion, respectively, including one patient who relapsed with CD19-negative blasts. One patient, who achieved morphological CR but had persistent BCR-ABL positivity, underwent allogeneic HSCT but relapsed 12 months after CAR-T infusion and 8 months post-transplant. The role of allogeneic HSCT following CAR-T-induced remission in B-ALL remains a subject of ongoing debate. In our setting, the principal challenge is timely identification and procurement of suitable donors. Notably, one patient who achieved CR experienced a CD19-negative relapse after a prolonged delay in undergoing allo-HSCT and ultimately died from the relapse without receiving the transplant. In our cohort, most CAR-T responders lacked access to suitable donors. Similarly low rates of allogeneic HSCT following CAR-T therapy in B-ALL (18–22%) have been reported in other studies [6, 9].

In our B-NHL cohort (*n* = 21), a high overall response rate (ORR) of 81% (*n* = 17) was observed as early as 1 month post-infusion, including a CR rate of 57.1% (*n* =  12). Two patients who initially had stable disease (SD) subsequently converted to CR at 5 and 11 months post-infusion, respectively. The best ORR achieved was 90.5% (*n* = 19). At a median follow-up of 12 months, 52.4% (*n* = 11) of patients remained in CR. These response rates are comparable to those observed in a registration trial of Tisa-Cel, with reported ORRs of 53% and CRs of 39% [3].

At median follow-ups of 12 and 36 months, durable responses were achieved in 57.1% of B-NHL patients treated with PP CAR-T, comparable to 60.4% reported with Tisa-cel in the JULIET study [3]. Of note, at a median follow-up of 12.5 months, the median overall survival was not reached in both our B-ALL and B-NHL cohorts.

However, approximately one-third of B-NHL patients who initially responded experienced relapse (*n* = 1, 4.8%) or disease progression (*n* = 6, 28.6%), primarily within the first 3 months following infusion. Additionally, two patients who did not achieve an initial response experienced early disease progression. These findings are consistent with previously reported relapse and progression rates of 30–60% within the first year after CAR-T therapy [10].

Among the five patients who relapsed or progressed after PP CAR-T infusion, most exhibited high-risk features such as elevated baseline LDH, primary extranodal disease, and adverse molecular subtypes (e.g., double- or triple-expressor DLBCL). All of these patients eventually died, with a median overall survival of 8.5 months. Notably, previous studies have reported a median survival of less than six months following CAR-T failure [9]. These observations underscore the urgent need for improved strategies to enhance outcomes in high-risk patients undergoing CAR-T therapy.

A robust in vivo expansion of CAR-T cells is strongly correlated with achieving CR [1, 4]. In our cohort, peak CD19+CAR-T cell levels exceeding 10% were observed in 87.5% (7 of 8) of B-ALL patients (range: 13.7–69.61%) and in 28.6% (6 of 21) of B-NHL patients (range: 13.38–47.03%), all of whom attained an early CR. In B-ALL, peak CAR-T expansion occurred between days 7 and 14 post-infusion, whereas in B-NHL, peak CAR-T levels were observed between days 7 and 21. Among the four B-ALL patients who relapsed, CD19+CAR-T cells were undetectable by day 21 in three cases. Likewise, all B-NHL patients who experienced relapse or disease progression had CAR-T cell levels below 1% on days 21 and 28. These findings are consistent with previous reports linking insufficient CAR-T cell persistence or rapid clearance to early treatment failure [1, 4].

In conclusion, PP CD19 CAR-T therapy offers a potentially life-saving option for patients with relapsed or refractory B-ALL and B-NHL who have exhausted standard therapies. Beyond clinical efficacy, this approach addresses significant logistical barriers in resource-limited settings. Local manufacturing shortens production timelines—crucial for patients with aggressive disease—and eliminates the need for overseas shipping, thereby reducing costs while preserving safety and efficacy that appear comparable to commercial CAR-T products. Nevertheless, larger studies with extended follow-up are required to confirm and further characterize these preliminary yet promising outcomes with this locally developed CAR-T cell therapy.

## Supplementary information

Supplementary Materials
