# Supplementary material for: Clinical outcomes of a new local CD19 CAR-T cell therapy for patients with relapsed or refractory acute lymphoblastic leukemia and non-Hodgkin lymphoma in Malaysia
Source: Bone Marrow Transplant. 2025 May 27;60(8):1199–203. doi: 10.1038/s41409-025-02629-8 (PMC12321574; doi:10.1038/s41409-025-02629-8)
Supplement: Supplementary file 1 — Supplementary Materials [file 41409_2025_2629_MOESM1_ESM.pdf]

Supplementary material

Summary

Supplementary Table S1: Basic features of PP CAR-T cells..........................1

Materials And Methods.. 1

Supplementary Figure S1 : Recruitment outcome... 11

Supplementary Table S1: **Basic features of PP CAR-T cells**

**Description**	**Characteristics and release criteria**
CAR-T generation	Second
Target (scFv)	CD19
Transmembrane protein	CD8α
Co-stimulatory domain	4-1BB
Signaling domain	CD3ζ
Final formulation	Fresh, 100ml
Dose	2 million CAR-T cells/Kg bodyweight
CD4/CD8 ratio	Donor dependent
T-cells selection method	Magnetic beads
Transduction method	Lentiviral
Safety (QC Testing)	Adventitious virus: Negative<br>Sterility testing: Negative<br>Mycoplasma: Absent<br>Endotoxin: <1.0 EU/mL<br>Replication Competent Lentivirus (RCL): Absent
Purity (QC Testing)	CD3+>70%<br>CAR19+ CAR-T/CD3+>20%<br>Residual beads: <100 beads per 3 million cells
Viability (Trypan blue exclusion method)	>70%

Materials And Methods

Study Design

Two prospective, parallel, open-label, single-arm phase II clinical trials were conducted between 2019 and 2024 at Pusat Terapi Sel, Hospital Canselor Tuanku Muhriz,

Universiti Kebangsaan Malaysia (HCTM-UKM). These trials involved patients with B-cell acute lymphoblastic leukemia (B-ALL) and B-cell non-Hodgkin lymphoma (B-NHL), who were treated with autologous CD19-41BB CAR-T cells (hereafter referred to as PP CAR-T cells). Both studies were approved by the UKM Research Ethics Committee (B-ALL: JEP-2019-003; B-NHL: JEP-2021-224) and registered at ClinicalTrials.gov (B-ALL: NCT03937544; B-NHL: NCT06698484). Written informed consent was obtained from all participants

Study Participants

Patients aged 13 to 75 years with relapsed /refractory B-ALL and B-NHL patients, with no available curative options such as hematopoietic stem cell transplantation (HCT), or had relapsed after HSCT were screened. All participants must have adequate organ function, defined as creatinine clearance > 50 ml/min, serum total bilirubin < 5 times the upper limit of normal, and left ventricular ejection fraction (LVEF) > 40%, ECOG performance status of ≤ 2, a life expectancy of over 3 months, and, for post-allogeneic HCT B-ALL patients, being ≥ Day +100 with no active GVHD and no ongoing immunosuppression. Female patients of childbearing potential must have a negative pregnancy test and use highly effective contraception methods, while male patients must also use highly effective contraception. Exclusion criteria include the presence of CNS involvement, active cancer other than B-ALL or B-NHL, severe comorbidities (including severe lung, heart, liver, or renal failure, and severe neurologic disorders), active autoimmune disease or atopic allergy, HIV positivity, active Hepatitis B or C infection, uncontrolled sepsis, ongoing prednisolone > 1 mg/kg daily or equivalent, and recent chemotherapy or immunotherapy within the past 3-4 weeks. Patients who met the eligibility criteria would be subjected to an assimilation study.

Sample size calculation

Sample calculation is using sealed envelope calculator website;

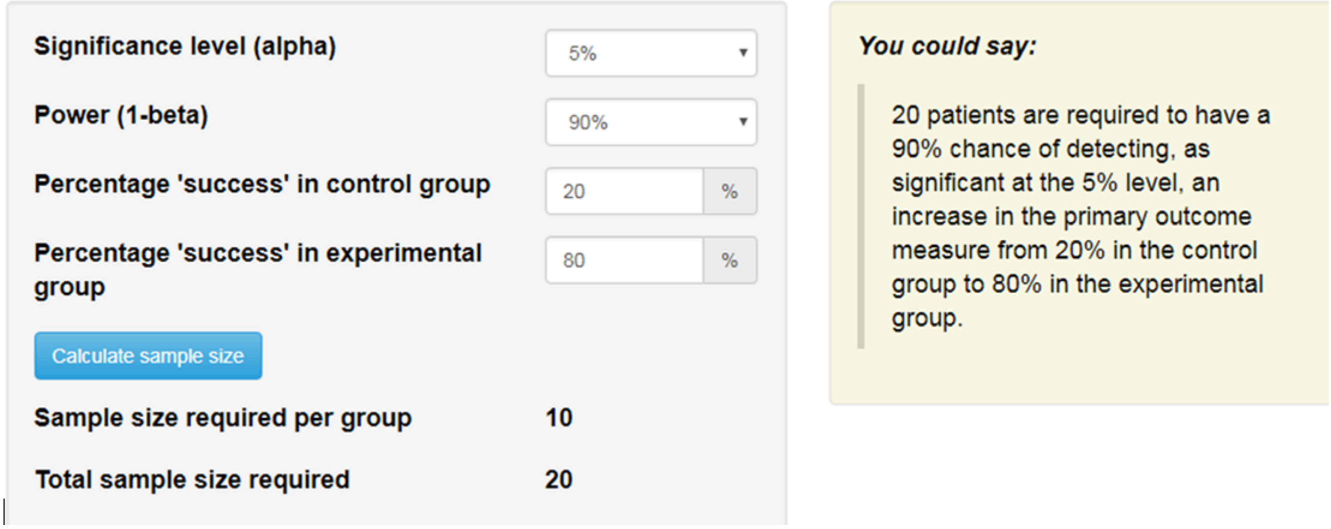

T cell Assimilation Test

The assimilation study serves as a critical pre-screening step in the CAR-T cell manufacturing process. Approximately 100–150 mL of peripheral whole blood is collected from clinical trial subjects to assess the suitability of their cells for CAR-T production.

During this study, mononuclear cells (MNCs) are isolated and cultured overnight (Day 0). T-cells are then selected and activated using magnetic bead-based selection. The activated T-cells are cultured further, and their expansion is evaluated on Day 6–7. On Day 0, flow cytometry is performed to determine the percentage of CD3+ cells among the MNCs. In parallel, a serology sample is collected to screen for adventitious agents such as HIV (Ab/Ag).

Subjects are considered to have passed the assimilation study if they test negative for adventitious agents and demonstrate good T-cell fitness.

T-Cell Fitness Criteria

Good T-cell fitness is defined by the following criteria:

- CD3+ cell percentage (Day 0): ≥ 3% of total MNCs
- Expansion fold (Day 6–7): ≥ 2-fold increase in cell count

T-cells that do not meet these benchmarks are considered to have poor fitness and may not be eligible for CAR-T manufacturing.

Patients who passed the T-cell assimilation test proceeded to apheresis at the stem cell facility in HCTM UKM, Kuala Lumpur. The collected cells were then transported to CryoCord's cGMP laboratory in Cyberjaya, located approximately 32 km from the apheresis site, for CAR-T cell manufacturing.

Apheresis Procedure

In this clinical trial, apheresis material is collected at the investigational site using standard apheresis equipment, Spectra Optia (Terumo BCT, USA). The apheresis process generally takes 3 to 4 hours, corresponding to approximately 10–20 L of recirculated blood and generates approximately 100–200 mL of apheresis material collection. The apheresis material collection bag is then shipped at 4 to 30ºC to the central manufacturing facility using validated shipping containers, where it is processed to enrich for the T cell-containing PBMC fraction using density gradient centrifugation.

Manufacturing of PP-CAR-T Cells

The manufacturing of autologous CD19 CAR-T (PP-CAR-T) cells was conducted in a cGMP grade B cleanroom at Plutonet's appointed contracted manufacturing organization (Cryocord, Malaysia) under current Good Manufacturing Practice (cGMP) conditions. This facility is certified according to the *Guidance Document and Guidelines for Registration of Cell and Gene Therapy Products (CGTPs)* [1], and the *PIC/S Guide to GMP for Medicinal Products* and its annexes [2]. Additionally, the cleanroom adheres to Biosafety Level 2 (BSL-2) standards for Living Modified Organisms (LMOs) in compliance with Malaysia's Biosafety Act [3].

To ensure the highest quality cellular products for human transfusion, all processing and production steps undergo comprehensive risk assessment, including risk identification, classification, and control measures. Controlled variables have been established at various stages to ensure consistent manufacturing. Moreover, extensive in-process and release testing—including assays for cell viability, sterility, mycoplasma, endotoxins, phenotyping, and serology screening—are conducted to confirm the safety and quality of the autologous CAR-T cells.

The CAR-T cell manufacturing process was initiated two weeks before the planned infusion, following an established protocol [4]. Briefly, autologous mononucleated cells (MNCs) were separated from the apheresis product. and cryopreserved in 10% dimethyl sulfoxide (DMSO) and stored at -150°C in the vapor phase of a liquid nitrogen storage tank.

MNCs were selected and activated using magnetic beads coated with anti-CD3/CD28 antibodies (Dynabeads®, Gibco, Lithuania). This activation initiated T-cell expansion and prepared the cells for transduction. After two days of activation, the T-cells were transduced with a lentiviral vector encoding a synthetic receptor. This receptor consisted of an extracellular single-chain antibody variable fragment (scFv) linked via a transmembrane domain (CD8α) to costimulatory (4-1BB) and CD3ζ signaling domains. The transduction was performed at a multiplicity of infection (MOI) ratio of 1:1.

Following transduction, PP-CAR-T cells were expanded in X-VIVO 15 media (Lonza Bioscience, USA) supplemented with 5% human AB serum (MP Biomedicals, USA) and recombinant interleukin-2 (500 IU/mL; R&D Systems, USA) for 10 days to achieve the therapeutic dose. The whole manufacturing process spans 14 days, during which samples were collected for quality control (QC) testing. Key assessments, such as transduction efficiency were performed on day 7 of culture. CAR-T cells were either infused fresh (n=24) or cryopreserved (n=6) prior to infusion in selected cases, due to clinical circumstances where CAR-T could not be administered on the planned date.

On the day of infusion, PP-CAR-T cells were harvested, washed, and resuspended in 100 mL of sodium chloride saline. The cell suspension was transferred into an infusion bag (JMS, Singapore), and additional samples were taken for final QC testing. Only CAR-T cell products that meet stringent quality standards, including sterility, endotoxin levels, and mycoplasma testing, were released for clinical use. This tightly controlled manufacturing process ensures the safety, consistency, and therapeutic efficacy of the PP-CAR-T cellular product.

CAR-T Cell Infusion Protocol

Following apheresis, bridging therapy was administered to over half of the B-ALL

(62.5%) and B-HNL (72.7%) patients to control the disease while awaiting the manufacturing of CAR-T cells. All patients received lymphodepleting chemotherapy with fludarabine (25 mg/m²/day IV) and cyclophosphamide (250 mg/m²/day IV) on Days -5, -4, and -3 prior to cell infusion to create an optimal environment for CAR-T cell expansion and cytotoxic activity. On Day 0, patients received a single dose of intravenous CD19 CAR-T cells at a target dose of 2 x 10^6 cells/kg.

A subsequent or repeated intravenous autologous CD19 CAR-T cell infusion at 3 or 6 months may be considered at the physician's discretion if the patient develops early loss of CAR-T cells with a significant B-cell recovery burden.

Measurement of CAR-T Cell Levels and Cytokines in Peripheral Blood

Following CAR-T cell infusion, blood samples were collected at multiple time points—Day 0, Day 7, Day 14, Day 21, and Day 28 to monitor CAR-T cell levels and inflammatory cytokine profiles were measured at post-infusion. For this purpose, 3 mL of peripheral blood was drawn into EDTA tubes for CAR-T cell level analysis, while 10 mL was collected in SST tubes for inflammatory cytokine analysis.

Flow cytometry was employed to measure circulating CAR-T cell levels post-infusion. Approximately 100 µL of peripheral blood was stained with fluorochrome-conjugated CD3 (BD Bioscience, USA) and CD19-CAR (proprietary detection reagent, YAKE Biotechnology, China) antibodies . The samples were incubated at 4°C for 20 minutes. Following incubation, a lysing buffer (BD Pharm Lyse) was added, and the samples were incubated at room temperature for 15 minutes. The samples were then washed twice with PBS, and cells were acquired using FACSLyrics (BD Bioscience, USA). Dead cells were excluded based on light scatter properties, and CAR-T cell levels were defined as lymphocyte/7-AAD-/CD3+/CD19CAR+ populations. In all patients, the number of CAR-T cells in peripheral blood was measured at multiple time points—Day 0, Day 7, Day 14, Day 21, and Day 28 post-infusion—to monitor cell expansion and persistence.

For cytokine analysis, 10 mL of peripheral blood was centrifuged to isolate serum. Cytokines, including IL-1β, IL-6, IL-8, IL-10, IL-12p70, and TNF, were measured using flow cytometry with the Human Inflammatory Cytokine Cytometric Bead Array (CBA) Kit (BD CBA I Kit, BD Bioscience, USA). IFN-γ levels were quantified via ELISA using the Human IFN-γ ELISA Kit (Biolegend, ELISA Max Deluxe).

These analyses provide critical data on CAR-T cell expansion, persistence, activity, and immune-related responses post-infusion, which are essential for evaluating therapeutic efficacy and managing potential adverse effects.

Assessment of Endpoints

The primary efficacy endpoint of the study was the overall response rate (ORR), defined as the proportion of subjects achieving either a partial response (PR) or a complete response (CR). Secondary endpoints included relapse rate, overall survival (OS), measured from the time of CAR-T cell infusion to the date of death, progression-free survival (PFS), measured from infusion to the date of disease progression, and duration of response (DoR).

Evaluations were conducted at study entry, followed by assessments starting from 4 to 6 weeks after CAR-T cell infusion. Subsequent evaluations occurred every 3 months during the first year, every 6 months during years 2 and 3, and annually thereafter. For patients with B-NHL, tumor response was assessed using Positron Emission Tomography-Computed Tomography (PET-CT) based on the Lugano classification [5]. For patients with B-ALL, bone marrow aspiration and trephine biopsy (BMAT) were performed to assess response, with minimal residual disease (MRD) measured using multicolor flow cytometry for Philadelphia chromosome (Ph)-negative B-ALL or real-time quantitative PCR (RQ-PCR) for BCR-ABL in Ph-positive B-ALL. Morphologic complete remission (CR) in B-ALL was defined as less than 5% bone marrow blasts, while MRD negativity was defined as less than 10^{-4} leukemic cells using multiparameter flow cytometry for Ph-negative patients or the absence of BCR-ABL transcripts by RQ-PCR in Ph-positive patients [6].

Safety endpoints focused on the incidence and severity of adverse events (AEs), graded according to the NCI Common Terminology Criteria for Adverse Events (CTCAE), version 4.0 [7], with special attention to cytokine release syndrome (CRS) and immune effector cell-associated neurotoxicity syndrome (ICANS). CRS and ICANS were graded based on the American Society for Transplantation and Cellular Therapy (ASTCT) consensus guidelines for cytokine release syndrome and neurologic toxicity associated with immune effector cells [8] .

At the 3-month post-infusion assessment, patients could choose to proceed with consolidative hematopoietic cell transplantation (HCT) or continue under surveillance based on discussions with their physician.

Study Oversight

The study was approved by the UKM Research Ethics Committee and conducted in compliance with the Declaration of Helsinki and Good Clinical Practice (GCP) guidelines. Written informed consent was obtained from all participants prior to enrollment. Oversight was provided by the Plutonet Data and Safety Monitoring Committee, which ensured the integrity and safety of the study. All authors reviewed and approved the final manuscript, confirming the accuracy and completeness of the data presented.

Statistical Analysis

Descriptive statistics were used to summarize baseline characteristics and clinical outcomes. Continuous data were expressed as median (range) while categorical variables were presented as frequency (%). The duration of response was calculated from the date of response or remission to the date of disease relapse or progression, while overall survival was determined from the day of CAR-T infusion to the date of death from any cause.

References

1. Ministry of Health. Guidance Document and Guidelines for Registration of Cell and Gene Therapy Products (CGTPs) in Malaysia [Internet]. 2016 [cited 2025 Jan 2]. Available from: https://www.npra.gov.my/index.php/en/guideline-bio/1527314-guidance-document-and-guidelines-for-registration-of-cell-and-gene-therapy-products-cgtps in-malaysia-2.html
2. PIC/S. PIC/S Guide to GMP for Medicinal Products [Internet]. [cited 2025 Jan 2] Available from: https://picscheme.org/en/publications?tri=gmp#zone
3. Ministry of Natural Resources and Environment. Biosafety Guidelines Contained Use Activity Of Living Modified Organism [Internet]. 2012 [cited 2025 Jan 2]. Available

from:
https://www.biosafety.gov.my/assets/document/garis-panduan-biokeselamatan/7-garis-panduan-aktiviti-kegunaan-terkawal-lmo.pdf

4. Pan J, Yang JF, Deng BP, et al. High efficacy and safety of low-dose CD19-directed CAR-T cell therapy in 51 refractory or relapsed B acute lymphoblastic leukemia patients. *Leukemia*. 2017;31(12):2587-2593
5. Cheson BD, Fisher RI, Barrington SF, Cavalli F, Schwartz LH, Zucca E, et al. Recommendations for initial evaluation, staging, and response assessment of Hodgkin and non-Hodgkin lymphoma: the Lugano classification. J Clin Oncol. 2014 Sep 20;32(27):3059–68.
6. Logan AC. Measurable residual disease in acute lymphoblastic leukemia: How low is low enough?. Best Practice & Research Clinical Haematology. 2022 Dec 1;35(4):101407.
7. Mendoza TR, Dueck AC, Bennett AV, Mitchell SA, Reeve BB, Atkinson TM, et al. Evaluation of different recall periods for the US National Cancer Institute's PRO-CTCAE. Clin Trials. 2017 Jun;14(3):255–63.
8. Lee DW, Santomasso BD, Locke FL, Ghobadi A, Turtle CJ, Brudno JN, et al. ASTCT consensus grading for cytokine release syndrome and neurologic toxicity associated with immune effector cells. Biol Blood Marrow Transplant. 2019 Apr;25(4):625–3

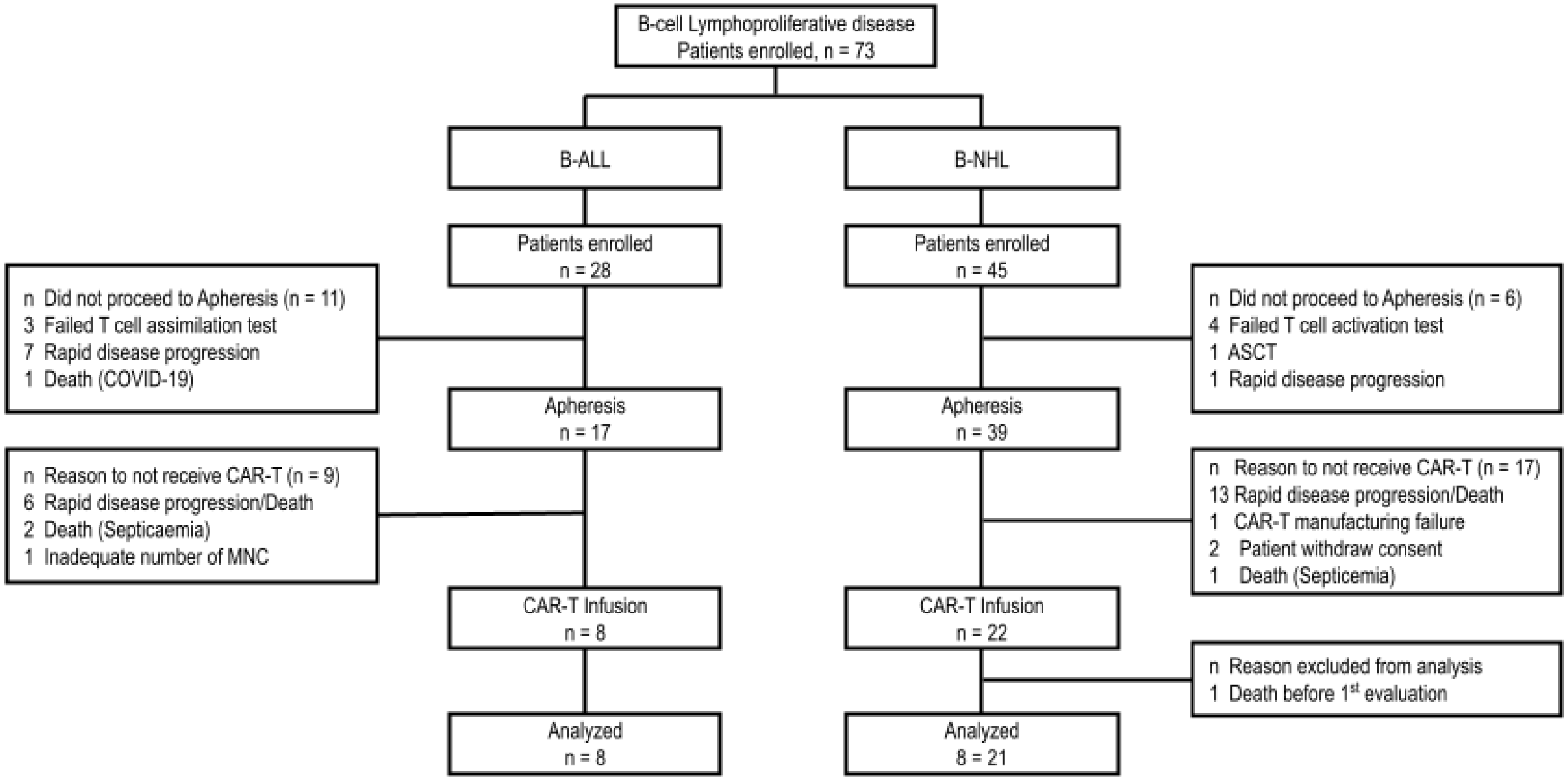

Supplementary Figure S1 : Recruitment outcome
